# Investigation of Bioactive Complexes of Chitosan and Green Coffee Bean or Artichoke Extracts

**DOI:** 10.3390/molecules28145356

**Published:** 2023-07-12

**Authors:** Deimante Rosliuk, Valdas Jakstas, Liudas Ivanauskas, Dovile Liudvinaviciute, Veronique Coma, Ramune Rutkaite

**Affiliations:** 1 Department of Polymer Chemistry and Technology, Kaunas University of Technology, Radvilenu Rd. 19, LT-50254 Kaunas, Lithuania; deimante.rosliuk@gmail.com (D.R.); dovile.liudvinaviciute@ktu.lt (D.L.); 2 Department of Pharmacognosy, Lithuanian University of Health Sciences, Sukileliu Ave. 13, LT-50162 Kaunas, Lithuania; valdas.jakstas@lsmu.lt; 3 Institute of Pharmaceutical Technologies, Lithuanian University of Health Sciences, Sukileliu Ave. 13, LT-50162 Kaunas, Lithuania; 4 Department of Analytical and Toxicological Chemistry, Lithuanian University of Health Sciences, Sukileliu Ave. 13, LT-50162 Kaunas, Lithuania; liudas.ivanauskas@lsmu.lt; 5 Laboratoire de Chimie des Polymères Organiques, Université de Bordeaux, CNRS, Bordeaux INP, UMR 5629, 16 Avenue Pey-Berland, F-33600 Pessac, France; veronique.coma@u-bordeaux.fr

**Keywords:** chitosan, artichoke extract, green coffee bean extract, caffeoylquinic acid, adsorption, antioxidant activity, antifungal activity

## Abstract

The formation of water-insoluble complexes between chitosan (ChS) and caffeoylquinic acid (CQ) derivatives present in artichoke (AE) and green coffee bean (GCBE) extracts was investigated by the equilibrium adsorption method. The UPLC/HPLC analysis revealed that the phenolic compounds accounted for 8.1% and 74.6% of AE and GCBE respectively, and CQ derivatives were the predominant compounds. According to the applied Langmuir adsorption model, anionic compounds present in natural extracts were adsorbed onto the active centers of ChS, i.e., primary amino groups. The driving forces of adsorption were electrostatic interactions between cationic groups of ChS and anionic compounds of natural extracts. Chromatographic analysis revealed that not only CQ derivatives, but also other phenolic compounds of natural extracts were attached to ChS. The release of adsorbed compounds into different media as well as the bioactive properties of complexes were also studied. With the immobilization of bioactives onto ChS, increased and prolonged ABTS^•+^ radical scavenging activity and decreased antifungal activity against *Fusarium graminearum* and *Botrytis cinerea* were observed compared to those of ChS. The findings of the current study highlight that the adsorption approach could be used to successfully prepare water-insoluble complexes of ChS and components of natural extracts with prolonged antioxidant activity.

## 1. Introduction

Caffeoylquinic acids are an important group of polyphenols in the human diet. These acids are the main derivatives of chlorogenic acid (an ester of caffeic and quinic acids) which is found in coffee, fruits, and vegetables, i.e., green coffee beans, cherries, artichoke, apricots, peaches, etc. [1,2,3]. There are three derivatives (isomers) of caffeoylquinic acids (CQ) which have one caffeoyl group, namely, 3-*O*-caffeoylquinic acid (chlorogenic acid), 5-*O*-caffeoylquinic acid (neo-chlorogenic acid) and 4-*O*-caffeoylquinic acid (crypto-chlorogenic acid) [4]. There are many literature data describing the studies on obtaining plant extracts containing high levels of CQ derivatives due to their high biological importance [5,6,7,8]. The biological effects of these compounds are mostly related to their antioxidant and anti-inflammatory activities [9,10]. Besides, among the health benefits attributed to CQ derivatives are a prevented risk of cardiovascular diseases and type 2 diabetes mellitus [11], and antibacterial and antiviral activity [12,13]. Lately, there has been a growing demand for chlorogenic acid due to its wide spectrum of applications in the food industry [14], pharmacology [15], and cosmetics industry [16]. However, the use of CQ derivatives is limited by their low bioavailability [17,18], high vulnerability to oxygen [19], or thermal processing [20]. To overcome these disadvantages, several immobilization methods have been described to ensure the protection and stability of chlorogenic acid such as extrusion, liposome entrapment, fluidized bed coating, spray drying, spray cooling/chilling, coacervation, centrifugal suspension separation, lyophilization, co-crystallization and emulsion formation [21,22,23,24].

Adsorption of CQ derivatives is an interesting attempt to improve stability under ambient conditions. The selection of adsorbent is an important factor in determining adsorption efficiency. Several adsorbents have already been tested in the adsorption of phenolic acids, such as cationic cross-linked starches with quaternary ammonium groups or tertiary amino groups [25,26]. Chitosan, a positively charged polysaccharide, is the N-deacetylation product of chitin that is mostly found in the exoskeleton of crustaceans, insects, and fungi [27]. The backbone of this copolymer consists of two repeating units, i.e., N-acetyl-2-amino-2-d-glucopyranose and 2-amino-2-deoxy-d-glucopyranose which are linked by β-(1 → 4)-glycosidic bonds [28]. Chitosan contains primary amines and hydroxyl groups which may be protonated in acid media and give a strong electrostatic attraction for anionic molecules such as metal ions (Ni^2+^, Hg^2+^, Cu^2+^, Pb^2+^) [29,30], organic dyes (Reactive Red, Acid Blue, Methyl Orange) [31,32] and pharmaceutical compounds (diclofenac, ibuprofen, ketoprofen) [33,34]. Recently, special attention has been paid to the development of biopolymers—bioactive materials with multi-functional delivery systems with targeted transfer and release of active compounds [35,36]. Due to abundant availability, high biocompatibility, excellent biodegradability, ability to form films, and especially polyelectrolyte behavior, chitosan could be also used as an adsorbent of anionic phenolics. The polyelectrolyte complexes are formed due to electrostatic interactions between the opposite counter ions. Furthermore, the complexes of chitosan—bioactive compounds have attracted a lot of attention, since obtained complexes exhibited advantageous properties of both components [37,38,39]. For example, chitosan nanoparticles prepared via ionic gelation were used for encapsulation of chlorogenic acid and showed enhanced antioxidant properties [39]. Apart from physical complexation, several grafting methods were employed to covalently attach the chlorogenic to chitosan [40,41]. For instance, the chitosan covalent complex with chlorogenic acid exhibited good thermal stability and antioxidant activity as well as the viscosity of the chitosan solution was increased after grafting of the chlorogenic acid [40]. Moreover, chlorogenic acid was successfully grafted onto chitosan using free radical grafting initiated by a hydrogen peroxide/ascorbic acid redox system to obtain derivatives with good antioxidant and antibacterial properties and the potential to be used as a postharvest fresh-keeping agent for fruits and vegetables [41]. However, the use of pure chlorogenic acid is expensive and the chemical modification route to prepare bioactive derivatives is also questionable due to the much longer preparation routine and changes in the properties such as the release of bioactives. Therefore, the adoption of natural extracts rich in chlorogenic acid derivatives as well as the use of simple complexation with suitable biopolymer adsorbents might be of great advantage when preparing tailored biobased materials with bioactive properties.

In the present study, the formation of water-insoluble complexes between chitosan and anionic phenolic compounds such as caffeoylquinic acid (CQ) derivatives, present in the artichoke (AE) and the green coffee bean (GCBE) extract, have been investigated. The adsorption on ChS was chosen as a method for the immobilization of active compounds of these natural extracts. The UPLC/HPLC procedures were used to monitor the composition of AE or GCBE as well as to estimate the quantities of active compounds adsorbed on ChS. The antioxidant and antifungal activities and release of active compounds from the formed complexes were studied.

## 2. Results

### 2.1. Chromatographic Analysis of AE and GCBE

**Figure 1:**
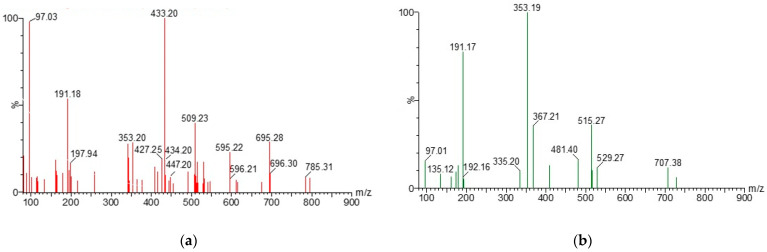
Mass spectra of AE (**a**) and GCBE (**b**).

The phenolic compounds of natural extracts were identified by the UPLC-ESI(-)-MS/MS method. MS–MS analysis of AE and GCBE (Figure 1) yielded the main ion at *m*/*z* 353, corresponding to caffeoylquinic (CQ) derivatives, i.e., chlorogenic, neo-chlorogenic, and crypto-chlorogenic acids. The peaks of the ion at *m*/*z* 515 and 191 were assigned to cynarine molecular ion which (Μ − H)^−^ *m*/*z* is 515 and fragment of quinic acid which *m*/*z* is 191. In the spectrum of AE the peak at *m*/*z* 447 also was observed and identified as a molecular ion of flavonoid luteolin 7-*O*-glucoside. In Figure 1b, the peak of an ion at *m*/*z* 367 was characterized as derivatives of ferulic and quinic acids, which has been identified by other researchers [42]. Moreover, a secondary fragment of coumaric derivatives at *m*/*z* 481 was determined. An analogous fragment (*m*/*z* 482.6–482.9) was also found by Alonso-Salcer et al. [43].

Dominant markers of AE and GCBE were selected for the UPLC-UV analysis. For quantitative analysis chlorogenic, neo-chlorogenic, and crypto-chlorogenic acids were monitored in the aqueous AE and GCBE solutions. Meanwhile, cynarine and luteolin 7-*O*-glucoside were also observed in AE solutions. The study was carried out under ultra-efficient liquid chromatography distribution conditions for the detection of eluent components using ultraviolet light absorption data at different wavelengths, namely, 325 nm for determination of chlorogenic, neo-chlorogenic, crypto-chlorogenic acids and total phenolic compounds, 321 nm—cynarine and 347 nm—luteolin 7-*O*-glucoside. The peak areas and the sum of peak areas were used for the quantitative estimation of individual compounds and total phenolic compounds. The results showed that AE and GCBE comprise 8.1% and 74.6% of the phenolic compounds, respectively. The other components of the extracts include carbohydrates, proteins, lipids, and minerals. Carbohydrates are the main component of artichoke extract and usually include simple sugars, disaccharides, polysaccharides, and some organic acids [44]. Furthermore, it was also found that AE contains 1.5% of chlorogenic acid, 0.6% of luteolin 7-*O*-glucoside, 0.5% of crypto-chlorogenic acid, 0.3% neo-chlorogenic acid (total amount of CQ derivatives being equal to 2.3%), and 0.2% of cynarine. Meanwhile, by testing the GCBE solution, it was found that chlorogenic acid, neo-chlorogenic acid, and crypto-chlorogenic acid embrace 32.5%, 6.6%, and 10.4% of the content, respectively (total amount of CQ derivatives being equal to 49.5%). Therefore, it is obvious that natural extracts under investigation contain quite different amounts of CQ derivatives.

### 2.2. Equilibrium Adsorption of AE and GCBE onto ChS

**Figure 2:**
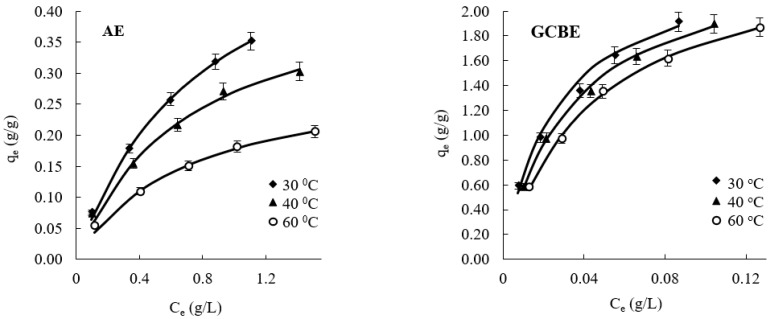
Adsorption isotherms of AE and GCBE onto ChS at different temperatures. Symbols represent experimental data and lines represent fitted curves of the Langmuir adsorption model.

As mentioned before, AE and GCBE contain 8.1% and 74.6% of phenolic compounds, respectively, and CQ derivatives are the predominant compounds, which comprise 2.3% and 49.5% of the total amount of phenolics, respectively. Chitosan having positively charged amino groups can form ionic complexes with negatively charged small molecules [45,46]. Therefore, ChS was used to test the adsorption of CQ derivatives as well as other anionic substances from aqueous solutions of AE and GCBE. It was found that adsorption equilibrium was achieved during the first 60 min. The obtained isotherms of AE and GCBE adsorption onto ChS at different temperatures are presented in Figure 2.

The Langmuir adsorption model [47] was used to describe the adsorption results (Figure 2). The main parameters of the Langmuir adsorption model calculated for the adsorption of AE and GCBE onto ChS, such as the Langmuir sorption capacity *Q_L_* and equilibrium constant *K_L_*, are given in Table 1 along with the values of the linear correlation coefficient *R*^2^. As could be seen from Table 1, the values of *R*^2^ > 0.99 support the fact that the adsorption data follows the used model. According to the Langmuir adsorption model, anionic compounds present in natural extracts were adsorbed onto the active centers of ChS, i.e., primary amino groups.

**Figure 3:**
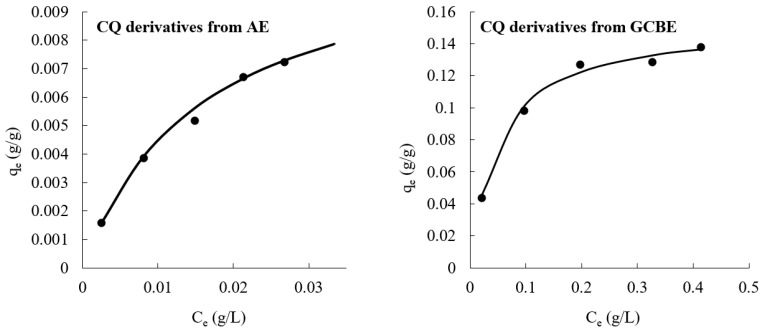
Adsorption isotherms of CQ derivatives adsorbed from AE and GCBE onto ChS at a temperature of 30 °C. Symbols represent experimental data and lines represent fitted curves of the Langmuir adsorption model.

The UV spectra absorbance maximum of the phenolics in AE and GCBE is the same as that of individual CQ derivatives, i.e., λ_max_ = 325 nm. Thus, the amount of adsorbed CQ derivatives on ChS particles is included in the amount of phenolics calculated from UV spectroscopy data. In order to determine the amounts of CQ derivatives attached to ChS, solutions after adsorption experiments at a temperature of 30 °C were analyzed by UPLC-UV-MS/MS. The obtained isotherms of CQ derivatives adsorption from AE and GCBE onto ChS are presented in Figure 3, and calculated parameters of the Langmuir adsorption model are presented in Table 2.

The UPLC-UV method was utilized to obtain more information about the changes in the composition of AE and GCBE solutions after the adsorption. During the interaction of the aqueous solution of AE with ChS powders, part of the bioactive compounds was adsorbed onto the polymer. It was established that 53.1% of chlorogenic acid, 52.1% of neo-chlorogenic acid, and 67.4% of crypto-chlorogenic acid were adsorbed. In the case of GCBE adsorption, it was found that 68.0% of chlorogenic acid, 74.3% of crypto-chlorogenic acid, and 76.4% of neo-chlorogenic acid were adsorbed onto the powder of ChS from the aqueous medium.

**Figure 4:**
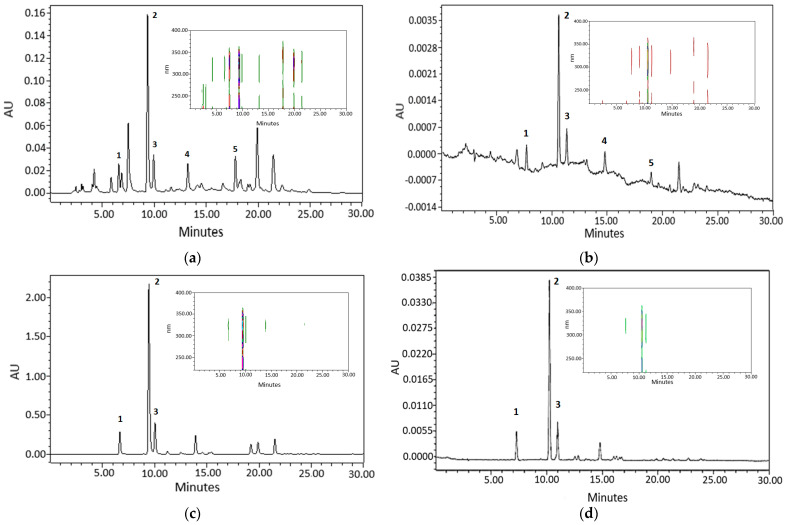
HPLC chromatograms at 325 nm wavelength and contour plots in the range of 200–400 nm of AE and GCBE: (**a**)—initial aqueous AE solution (contour plot scale 0.016–0.160 AU); (**b**)—aqueous AE solution after adsorption experiment (contour plot scale −0.0012–0.0035 AU); (**c**)—initial aqueous GCBE solution (contour plot scale 0.200–2.000 AU); (**d**)—aqueous GCBE solution adsorption experiment (contour plot scale 0.0002–0.0385 AU). Annotation of selected peaks: 1—neo-chlorogenic acid, 2—chlorogenic acid, 3—crypto-chlorogenic acid, 4—cynarine, 5—luteolin 7-*O*-glucoside.

Pharmaceutical and nutraceutical specifications of herbal ingredients and preparations include fingerprints as the main parameter of quality. Since the chromatograms alone do not provide information about spectral characteristics of eluted compounds that are related to chromophore functional groups there was a need to monitor photodiode-array detection data in the 200–400 nm spectral range. Monitoring fingerprint chromatograms at 325 nm was carried out and photodiode-array detector contour plots of AE and GCBE solutions were obtained using separation gradient according to the official European Pharmacopoeia monograph 1866. As could be seen in Figure 4, the retention time of neo-chlorogenic acid was about 7 min, chlorogenic acid—9.5 min, and crypto-chlorogenic acid—10 min. The contour plots of photodiode-array detection data of AE and GCBE demonstrate a predominance of compounds whose spectra have maxima in the range of 270–350 nm and are related to phenolic chromophores. The fingerprint chromatograms and plots of solutions before and after adsorption demonstrate changes in both concentration and also a chromatographic profile of the bioactive solutes.

### 2.3. Release of AE and GCBE from Complexes

AE/ChS or GCBE/ChS powders having 0.116 g and 0.180 g of adsorbed AE and GCBE per 1 g of ChS, respectively, were chosen for the assessment of release of phenolics from formed complexes into different media, namely, 0.1 mol/L HCl solution (model stomach medium), 0.1 mol/L acetate buffer pH = 4.1, 0.1 mol/L phosphate buffer pH = 6.8 (model intestinal medium) and distilled water. The release of phenolic compounds from ChS complexes was examined using UV absorbance at 325 nm data. As can be seen from data presented in Table 3 the amount of released AE or GCBE phenolics depends on the release medium.

It was found that in 30 min, AE/ChS and GCBE/ChS complexes powders were completely dissolved in 0.1 mol/L HCl solution because ChS is readily soluble in acidic solutions [48]. Similarly, AE/ChS complex powder was also dissolved in acetate buffer (pH = 4.1). Meanwhile, 92.8% of GCBE phenolics were released from the complexes into 0.1 mol/L acetate buffer. The difference in the solubility of complexes in acetate buffer solution could be associated with different amounts of CQ derivatives in natural extracts, i.e., GCBE contains 49.5% of CQ derivatives while AE only 2.3%. Moreover, 50.0% and 42.3% of AE and GCBE, respectively, were released from the complexes into 0.1 mol/L phosphate buffer at pH = 6.8. However, the release of phenolics from the complexes was insignificant in distilled water.

### 2.4. Bioactive Properties of Complexes

UPLC and HPLC analysis allows us to state that the composition of phenolics adsorbed onto ChS is different depending on the composition of AE and GCBE. This difference may result also in different antioxidant and antifungal activities of crude extracts and complexes.

**Figure 5:**
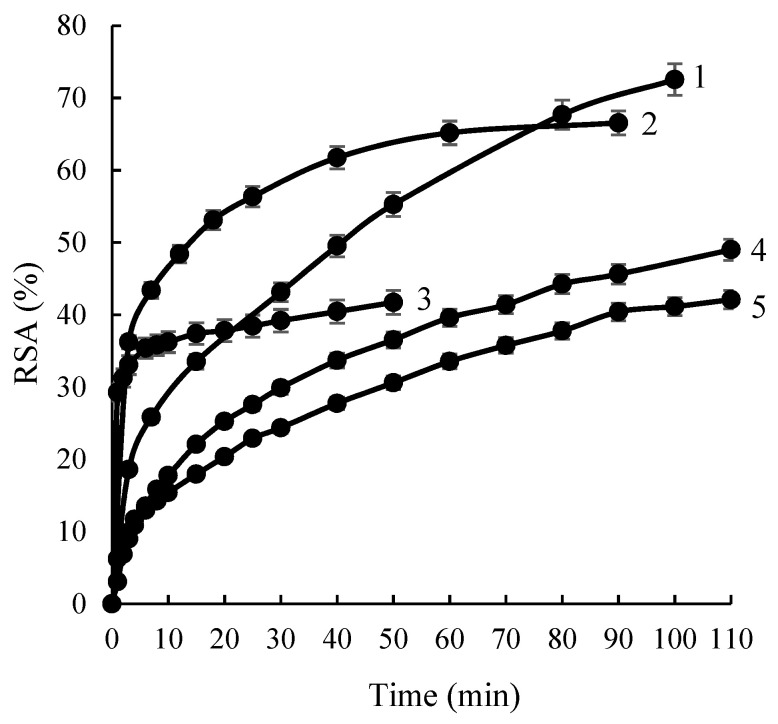
Time dependent ABTS^•+^ radical scavenging activity (*RSA*): 1—GCBE/ChS; 2—GCBE; 3—AE; 4—AE/ChS; 5—ChS. Concentration of extracts and those present in AE/ChS or GCBE/ChS in ABTS^•+^ solution was the same and equal to 0.0038 g/L.

Antioxidant activity assessment. The antioxidant activity of AE/ChS and GCBE/ChS was evaluated by the ABTS^•+^ method and expressed as time dependent ABTS^•+^ radical scavenging activity (*RSA*,%) (Figure 5). The antioxidant activity of initial AE and GCBE solutions also was determined. The amount of natural extracts in the individual AE and GCBE solutions, and those containing AE/ChS and GCBE/ChS powders was the same. As seen from the data provided the antioxidant activity of phenolic compounds linked to ChS is higher in comparison with the antioxidant activity of AE and GCBE (curves 1 and 4). It could be assumed that the dominance of CQ derivatives in AE/ChS and GCBE/ChS powders also determined their higher antioxidant activity. It is known that chlorogenic, neo-chlorogenic, or crypto-chlorogenic acids having five hydroxyl groups possess a great impact on antioxidant efficiency.

Moreover, the immobilization of phenolic compounds of extracts prevented the rapid loss of antioxidant activity, i.e., AE/ChS and GCBE/ChS powders exhibited prolonged radical scavenging. It should be noted that chitosan itself possesses good antioxidant activity (*RSA*~41%, Figure 5, curve 5) due to its unique properties. Moreover, the antioxidant activity of complexes could also depend on the amount of adsorbed CQ derivatives and the amount of CQ derivatives adsorbed from GCBE is 15 times higher in comparison to that from AE (Table 2). Besides, UPLC and HPLC analysis data showed that not only are CQ derivatives adsorbed from AE and GCBE but other phenolic compounds also are, which might affect the antioxidant activity of the complexes.

Antifungal activity studies. For antifungal activity tests, *B. cinerea* and *F. graminearum* were selected and the antifungal activity of AE/ChS and GCBE/ChS was compared to that of ChS. Clearly, the fungal growth of both species was reduced by using both complexes; however, the calculated inhibition of growth values were still lower than those determined for ChS powder, especially compared to GCBE/ChS (Table 4).

Indeed, AE/ChS showed similar antifungal activity when compared to pure ChS, with 50% and 16% inhibition against *B. cinerea* and *F. graminearum*, respectively. In the case of GCBE, 2–3 times lower inhibition of growth values was determined. This result suggests that CQ derivatives adsorbed on ChS act not as antifungal agents but, in contrast, may even increase the growth of both fungi to some extent.

## 3. Discussion

Water-insoluble complexes have been formed between ChS and CQ derivatives present in AE and GCBE by the equilibrium adsorption method. The UPLC/HPLC analysis revealed that AE and GCBE contained 8.1% and 74.6% of phenolic compounds, respectively, and CQ derivatives were predominant compounds which comprised 2.3% and 49.5% of the total amount of phenolics, respectively.

The Langmuir model has been applied to describe the equilibrium adsorption data.

It was found that Langmuir sorption capacity values *Q_L_* for GCBE adsorption onto ChS were about 4–8 times higher than those for AE adsorption onto ChS at all applied temperatures. One of the reasons for such differences could be the higher content of caffeoylquinic acid derivatives in GCBE. Moreover, it was determined that the driving forces of adsorption were electrostatic interactions between cationic groups, i.e., primary amino groups of ChS and anionic compounds of natural extracts. The obtained adsorption data are comparable to the results of our previous study [6] where phenolic compounds of these natural plant extracts were adsorbed on the quaternary ammonium groups of cross-linked cationic starch microgranules.

The comparison of the values of Langmuir sorption capacities *Q_L_* calculated for the adsorption of total phenolics from UV spectroscopy data and those quantified for adsorption of CQ derivatives by chromatography, revealed that the content of adsorbed CQ derivatives was only 1.6% and 6.1% of the total amount of adsorbed phenolics from AE and GCBE, respectively. The obtained results indicated that not only CQ derivatives, but also other unidentified phenolic compounds of natural extracts could be immobilized onto ChS. It can be presumed that other phenolics were competing with CQ derivatives during adsorption. It is known that this competitive adsorption might be dependent on the steric hindrance and different affinities of phenolic compounds of the extracts [49].

CQ derivatives and other phenolics could be released from the obtained complexes into different media. ABTS^•+^ radical scavenging activity and antifungal activity against *F. graminearum* and *B. cinerea* studies showed that the immobilization of bioactives onto ChS can have opposite effects.

The higher antioxidant activity of phenolic compounds linked to ChS was determined in comparison with the antioxidant activity of AE and GCBE. Similar results, i.e., lower antioxidant activity of pure AE and GCBE compared to that of cross-linked cationic starch and AE or GCBE complexes were observed in our previous study [6]. It is well known that chitosan itself possesses good antioxidant activity (*RSA*~41% in our study) due to its unique properties and this also might affect the activity of the complexes. These findings are consistent with those shown by Woo et al. [50], which observed an *RSA* of about 37% at the concentration of ChS equal to 0.05 g/L. Moreover, the antioxidant activity of complexes could also depend on both the amount of attached CQ derivatives which was higher in the case of GCBE, and the presence of additional phenolic compounds.

Although higher and prolonged antioxidant activity was observed in the case of complexes, a reduction in antifungal activity was observed when compared to ChS, especially in the case of GCBE/ChS. These results correlate with the work completed by Schöneberg et al. [51], where it was shown that some phenolic compounds such as p-hydroxybenzoic acid, vanillic acid, quercetin, and rutin have a slight stimulating effect on the mycelium growth of *F. graminearum*. That is also in correlation with the quantities of CQ derivatives adsorbed on ChS particles, as much higher amounts of CQ derivatives were attached to ChS from GCBE than from AE. It let us conclude that both type and quantity of phenolics adsorbed from natural extracts onto ChS are of great importance when testing antifungal activity. Thus, further studies are needed to evaluate the potential of developed bioactive materials in both food and food packaging applications.

## 4. Materials and Methods

### 4.1. Materials

The low molecular weight chitosan powder (ChS), 2,2′-azino-bis(3-ethylbenzothiazoline-6-sulphonic acid) (ABTS) were purchased from Sigma-Aldrich and used without further purification. The artichoke extract (AE) powder was supplied by Bernett S.R.L. Affiliate of Indena (Italy). The green coffee bean extract (GCBE) powder was received from ZD Biological (China). Reference analytical standards were purchased from Sigma Aldrich (USA) and their purity was certified using HPLC (neochlorogenic acid (98%), chlorogenic acid (95%), crypto-chlorogenic acid (98%), cynarine (98%), luteolin 7-*O*-glucoside (98%). Potato Dextrose Agar (PDA) medium (potato starch 4 g/L, dextrose 20 g/L, agar 15 g/L) was provided by Biokar. The *Fusarium graminearum* strain CBS 185.32 (Westerdijk Fungal Biodiversity Institute, previously Centraal bureau voor Schimmelcultures (CBS), The Netherlands) with the DON/15-ADON chemotype and a *Botrytis cinerea* strain isolated by INRAe (MycSA UR 1264 Mycologie and Sécurité des Aliments) from a vineyard in Aquitaine (France) were used throughout this study. All other reagents were of analytical grade and used without purification.

### 4.2. Equilibrium Adsorption Studies

0.1 g of ChS was placed into an Erlenmeyer flask, and 100 mL of AE and GCBE aqueous solutions of a certain concentration were added. The concentration of AE or GCBE aqueous solution varied from 0.1 to 1 g/L. The flask was stoppered and shaken for 60 min at temperatures of 30 °C, 40 °C, and 60 °C and fixed shaking intensity in a thermostating bath with the temperature control of ±1 °C (Memmert GmbH, Schwabach, Germany). Then the mixture was filtered through a paper filter, and the residual concentration of AE and GCBE in the filtrate solution was estimated. The amount of the adsorbed natural extracts *q_e_* (g/g) was calculated according to the equation:

(1) $$q_{e}=\frac{\left(C_{o}-C_{e}\right)\cdot V}{W}$$

where *C_o_* is the initial concentration (g/L) of AE or GCBE, *C_e_* is the concentration (g/L) at the equilibrium in the supernatant solution, *V* is the volume of the solution (L), and *W* is the weight of ChS (dry material, g).

To determine the concentration of AE or GCBE in the supernatant the UV absorbance at λ_max_ = 325 nm of the solution was measured by using a U*V/V*IS spectrophotometer Jenway 6715 (Bibby Scientific Ltd., Stone, UK). The concentration of AE or GCBE was determined from the calibration curves. AE and GCBE reference solutions were prepared in the concentration range of 0.025–0.5 g/L and 0.01–0.1 g/L, respectively. The correlation coefficient (*R*^2^) of generated calibration curves was 0.9997 and 0.9991 for AE and GCBE, respectively.

The Langmuir adsorption model [47] was used for fitting adsorption results. According to the Langmuir adsorption model [47], the adsorption takes place at specific homogeneous sites within the adsorbent, and once an adsorbate molecule occupies a site, no further adsorption can take place. The Langmuir equation may be presented as

(2) $$q_{e}=\frac{Q_{L}K_{L}C_{e}}{1+K_{L}C_{e}}\;\;(\mathrm{non}-\mathrm{linear}\;\mathrm{form})$$

where *q_e_* (g/g) is the amount of the adsorbate adsorbed by adsorbent at the equilibrium, *C_e_* (g/L) is the equilibrium concentration of the adsorbate, *Q_L_* (g/g) is the Langmuir sorption capacity, and *K_L_* (L/g) is the Langmuir equilibrium constant. When *C_e_*/*q_e_* versus *C_e_* was plotted, the value of *Q_L_* was calculated from the slope and the value of *K_L_* from the intercept.

### 4.3. Chromatographic Analysis

Separation of GCBE, AE, and supernatant solutions after the adsorption experiments were carried out with an Acquity H-class UPLC system (Waters Corporation, Milford, MA, USA) equipped with an Acquity BEH column (2.1 × 50 mm, 1.7 μm). Gradient elution was performed with 0.1% formic acid water solution (solvent A) and acetonitrile (solvent B) with the flow rate set to 0.4 mL/min. A linear gradient profile was used with the following proportions of solvent B: initial—2%, 0.5–2.0 min—15%, 4.0–5.5 min—50%, 5.5 min, 6.5 min—100%. The injection volume was 1 μL. Xevo TQD MS/MS detector (Waters Corporation, Milford, MA, USA) was used to obtain MS and MS/MS data. Negative electrospray ionization was used with the following settings: capillary voltage—1.5 kV, source temperature—150 °C, desolvation temperature—500 °C, desolvation gas flow—1000 L/h, cone gas flow—25 L/h. The identification of analytes was carried out using UPLC-MS/MS detection (MRM), and MS and UV spectra (obtained using an Acquity eLambda photodiode-array detector). For the detection of analytes UV light absorption at different wavelengths was chosen. Determination of chlorogenic, neo-chlorogenic, crypto-chlorogenic acids, and total phenolic compounds was made at 325 nm. The 321 nm wavelength was chosen for cynarine and 347 nm for luteolin 7-*O*-glucoside. Peak areas were used for quantitative processing, and the sum of peak areas of the chromatogram at 325 nm was used for estimating total phenolic markers. Linear Through Zero fit type calibration (peak area versus concentration) was chosen for analysis. Analytical standard solutions were prepared in the concentration range of 0.122–250 μg/mL. *R*^2^ of generated calibration curves ranged from 0.9997 (curves of neochlorogenic acid and luteolin 7-*O*-glucoside) to 0.9999.

A Waters 2695 chromatograph equipped with a Waters 2998 photodiode-array detector (Waters Corporation, Milford, MA, USA) was used for the monitoring of chromatographic profile according to the procedure of European Pharmacopoeia monograph 04/2018:1866. Data was managed with the Empower^®^ 3 program software (Waters Corporation, Milford, MA, USA). Chromatographic separations were carried out by using an ACE (5 μm, C_18_, 250 × 4.6 mm i.d.) column equipped with a pre-column. The volume of the extract being investigated was 10 μL. The flow rate was 1.2 mL/min. The mobile phase consisted of solvent A (phosphoric acid R, water R 0.5:99.5 *V*/*V*) and solvent B (phosphoric acid R, acetonitrile R 0.5:99.5 *V*/*V*). The following conditions of elution were applied: 0–1 min, 8% B; 1–20 min, 8–25% B; 20–33 min, 25% B; and 33–35 min, 25–100% B. The column was operated at a constant temperature of 40 °C. The chromatographic profile was monitored by comparing the retention times. Spectral characteristics (λ = 200–400 nm) of the eluted peaks were also monitored.

### 4.4. Preparation of Complexes

One gram of ChS was mixed with 250 mL of 1 g/L AE or GCBE aqueous solutions and stirred at 300 rpm for 60 min at room temperature (20 ± 1 °C). After adsorption, the mixture was filtered through a glass filter, the residual concentration of AE or GCBE in the filtrate was determined by UV spectroscopy as described in Section 4.2, and the quantities of AE or GCBE adsorbed on ChS were calculated. The obtained powders were washed with distilled water and dried at room temperature (20 ± 1 °C). ChS microgranules with adsorbed AE or GCBE were named as AE/ChS and GCBE/ChS, respectively. The prepared complexes were used for release, antioxidant, and antifungal activity studies.

### 4.5. Release Study

A total of 0.1 g of AE/ChS or GCBE/ChS were mixed with 50 mL of release medium, stirred at 300 rpm for 30 min at room temperature (20 ± 1 °C), and filtered through a glass filter. The AE and GCBE concentrations in the filtered solutions were estimated by using UV spectroscopy as described in Section 4.2. The percentage of released AE or GCBE was calculated as a ratio between the released amount of extract and the amount of extract added to the medium with AE/ChS or GCBE/ChS powder.

The distilled water, ethanol, 0.1 mol/L HCl solution, and 0.1 mol/L acetate buffer solution at pH = 4.1, as well as 0.1 mol/L phosphate buffer solution at pH = 6.8, were used as the release medium.

### 4.6. Antioxidant Activity Study

ABTS radical scavenging activity of AE/ChS, GCBE/ChS, AE, and GCBE was assessed by measuring the disappearance of the bluish-green color of the aqueous ABTS radical cation solution [52]. First of all, ABTS was dissolved in water to make a 7 mM solution. ABTS radical cation (ABTS^•+^) was produced by reacting ABTS stock solution with 2.45 mM potassium persulfate in equal quantities (*V*/*V*) and allowing the mixture to stand in the dark at room temperature for 12–16 h before use. As ABTS and potassium persulfate react stoichiometrically at a ratio of 1 to 0.5, this will result in incomplete oxidation of the ABTS. For the study of natural extracts and their complexes with ChS, the ABTS^•+^ solution was diluted with phosphate buffer solution pH 4.1, to adjust a light absorbance at 734 nm to 1 ± 0.02 a.u. A total of 0.00183 g of AE/ChS or 0.00125 g of GCBE/ChS dried powder was mixed with 50 mL of diluted ABTS^•+^ solution and stirred at 300 rpm at room temperature. At certain periods of time, the absorbance of ABTS^•+^/phosphate buffer solution at 734 nm was measured with a visible light spectrophotometer T60 Vis (PG Instruments Ltd., Wibtoft, UK). The ABTS^•+^/phosphate buffer solution free radical scavenging activity (*RSA*, in%) was calculated using the following equation [52]:

(3) $$\mathrm{RSA}=\frac{A_{t}}{A_{\mathrm{initial}}}\cdot 100\%$$

where *A_initial_* is the absorbance of the initial ABTS^•+^/phosphate buffer solution, and *A_t_* is the absorbance of ABTS^•+^/phosphate buffer solution containing the sample at the time *t*.

In the same way, experiments with AE and GCBE extracts were performed.

### 4.7. Antifungal Activity Study

When inoculum was required, fungal strains were grown on PDA at 25 °C for one week in the dark. Spores suspensions was prepared in CMC medium (15 g/L carboxymethyl cellulose, 1 g/L yeast extract, 0.5 g/L MgSO_4_.7H_2_O, 1 g/L NH_4_NO_3_, 1 g/L KH_2_PO_4_). Four agar blocks (5 mm in diameter) carrying mycelia were introduced into 40 mL of CMC medium and incubated in darkness at 25 °C and at 180 rpm. After 2–3 days, culture broths were filtered in order to remove the mycelia. Spores were precipitated after centrifugation (2300× *g* for 5 min) and washed two times with sterile distilled water. Spores were suspended in 2% Tween solution and concentration was determined using the spores counting method.

For antifungal activity tests, AE/ChS and GCBE/ChS powders having 0.104 g and 0.178 g of adsorbed AE and GCBE, respectively, per 1 g of ChS were used.

The antifungal activity of the samples (ChS, AE/ChS, and GCBE/ChS) against *B. cinerea* was evaluated by the growth inhibition bioassay. The ChS, AE/ChS, or GCBE/ChS were dispersed in a sterile culture medium (PDA) at the concentration of ChS in PDA being equal to 5 g/L. Following thorough mixing, the medium was poured into a Petri dish 85 mm in diameter. The 5 mm diameter agar plugs inoculated with fungi were incubated on prepared agar plates with the samples. Petri dishes were incubated at 25 °C at 70% relative humidity in the dark. The control condition corresponded to the PDA medium. The colony growth diameter was measured for six days. The experiments were performed in triplicate.

The antifungal activity of the samples (ChS, AE/ChS, and GCBE/ChS) against *F. graminearum* was evaluated by the growth inhibition bioassay. The ChS, AE/ChS, or GCBE/ChS were dispersed in a sterile culture medium (PDA) at the concentration of ChS in PDA being equal to 5 g/L. Following thorough mixing, the medium was poured into a Petri dish 85 mm in diameter. About 10 μL of *F. graminearum* spore suspension (containing 100 spores/mL), were inoculated on the center of the dish. Petri dishes were incubated at 24 °C at 70% relative humidity in the dark. The control condition corresponded to the PDA medium. The colony growth diameter was measured for five days. The experiments were performed in triplicate.

For both fungi, inhibition of growth (%) at day 4 was calculated using the following equation:

(4) $$Inhibition\;\left(\%\right)=\frac{d_{c}-d_{s}}{d_{c}}\cdot 100$$

where *d_c_* is the diameter of the hyphal extension zone of blank (control) (mm) and *d_s_* is the diameter of the hyphal extension zone of the tested sample (mm).

Inhibition values were expressed as mean values ± standard deviation.

## Figures and Tables

**Table 1:** Parameters of Langmuir model for AE and GCBE adsorption onto ChS at different temperatures.

*T* (°C)	*Q_L_* (g/g)	*K_L_* (L/g)	*R* ^2^
Adsorption of AE			
30	0.61	1.24	0.999
40	0.46	1.45	0.994
60	0.31	1.40	0.999
Adsorption of GCBE			
30	2.45	38.20	0.991
40	2.49	29.52	0.998
60	2.51	22.89	0.999

**Table 2:** Parameters of Langmuir model for CQ derivatives adsorption from AE and GCBE onto ChS at a temperature of 30 °C.

Natural Extract	*Q_L_* (g/g)	*K_L_* (L/g)	*R* ^2^
AE	0.01	62.94	0.999
GCBE	0.15	20.07	0.996

**Table 3:** Influence of the medium on the amount of AE and GCBE phenolics released from AE/ChS and GCBE/ChS.

Release Medium	Concentration ^1^ of Phenolics, (mg/L)/(%)	
	AE	GCBE
0.1 mol/L HCl (pH = 1)	Dissolved	Dissolved
Acetate buffer (pH = 4.1)	Dissolved	283/92.8
Phosphate buffer (pH = 6.8)	104/50.0	129/42.3
Distilled water (pH = 5)	13.3/6.4	13/4.3

^1^ concentration calculated considering the amount of extract added to the medium with AE/ChS and GCBE/ChS powder was C_AE_ = 208 mg/L and C_GCBE_ = 305 mg/L, respectively.

**Table 4:** Inhibition of growth of *F. graminearum* and *B. cinerea* on PDA medium supplemented with ChS, AE/ChS, and GCBE/ChS particles after four days of inoculation at 25 °C.

Sample	Concentration of Phenolics in PDA (g/L)	Concentration of ChS in PDA (g/L)	Inhibition of Growth at Day 4 (%)	
			*B. cinerea* *mycelium*	*F. graminearum* *spores*
ChS	-	5	54 ± 1	21 ± 1
AE/ChS	0.52	5	50 ± 1	16 ± 1
GCBE/ChS	0.89	5	37 ± 1	6 ± 1

## Data Availability

Not applicable.
